# Detection of divergent genes in microbial aCGH experiments

**DOI:** 10.1186/1471-2105-7-181

**Published:** 2006-03-30

**Authors:** Lars Snipen, Dirk Repsilber, Ludvig Nyquist, Andreas Ziegler, Ågot Aakra, Are Aastveit

**Affiliations:** 1 Biostatistics, Department of Chemistry, Biotechnology and Food Sciences, Norwegian University of Life Sciences, N-1432 Ås, Norway; 2 Department of Biology and Biochemistry/Bioinformatics, University of Potsdam, Germany; 3 Microbial Gene Technology, Department of Chemistry, Biotechnology and Food Sciences, Norwegian University of Life Sciences, Ås, Norway; 4 Institute of Medical Biometry and Statistics, University at Lübeck, Germany

## Abstract

**Background:**

Array-based comparative genome hybridization (aCGH) is a tool for rapid comparison of genomes from different bacterial strains. The purpose of such analysis is to detect highly divergent or absent genes in a sample strain compared to an index strain. Development of methods for analyzing aCGH data has primarily focused on copy number abberations in cancer research. In microbial aCGH analyses, genes are typically ranked by log-ratios, and classification into divergent or present is done by choosing a cutoff log-ratio, either manually or by statistics calculated from the log-ratio distribution. As experimental settings vary considerably, it is not possible to develop a classical discriminant or statistical learning approach.

**Methods:**

We introduce a more efficient method for analyzing microbial aCGH data using a finite mixture model and a data rotation scheme. Using the average posterior probabilities from the model fitted to log-ratios before and after rotation, we get a score for each gene, and demonstrate its advantages for ranking and detecting divergent genes with enlarged specificity and sensitivity.

**Results:**

The procedure is tested and compared to other approaches on simulated data sets, as well as on four experimental validation data sets for aCGH analysis on fully sequenced strains of *Staphylococcus aureus *and *Streptococcus pneumoniae*.

**Conclusion:**

When tested on simulated data as well as on four different experimental validation data sets from experiments with only fully sequenced strains, our procedure out-competes the standard procedures of using a simple log-ratio cutoff for classification into present and divergent genes.

## Background

The genetic diversity among bacteria mirrors their lifestyles and physiological versatilities and evolves from adaptation to their niches and growth conditions. Many techniques have been used to obtain a picture of true microbial diversity. Microarray-based comparative genome hybridization (aCGH) is now a commonly used tool in comparative genomics. Compared to sequencing and comparing whole genomes, aCGH provides rapid genomotyping in bacteria [1,2].

The majority of applications of aCGH is in cancer-research, where copy-number abberations is the primary focus [3,4]. Several methods have been suggested to analyze such data, e.g. [5-7].

In microbial studies of genome diversity, usually one fully sequenced strain, called index strain, is compared to a set of unsequenced strains of the same or closely related bacterial species, called sample strains. In this setting it is of interest to characterize the sample strains with respect to the genes they have in common with the index strain, and those which are absent or highly divergent.

In theory, every given gene is either present or divergent in the sample strain. In this respect, a perfect measurement technology would provide a binary output. For many reasons, this is not the case in aCGH. First, it is complicated to define relationships between sequence identity and hybridization signals. Second, hybridization signals arise both from hybridization with similar genes, as well as from hybridization with homologs, paralogs, or genes with conserved domains. Such non-specific hybridizations may lead to signals even from genes that are truly divergent. Third, gene divergence is a slow evolutionary process such that based on nucleotide sequence similarity alone, in most cases a number of genes will be difficult difficult to classify as divergent or present. Finally, the experimental features of aCGH may complicate the interpretation of the hybridization patterns.

Usually, the samples for microbial aCGH are prepared as follows: genomic DNA is extracted from the index and from the sample strain. The DNA is then physically sheared or enzymatically digested, and the resulting fragments are labelled with different fluorescent dyes by random priming. The labelled samples are mixed and then hybridized onto the microarray. In contrast to gene expression experiments, the preparation of samples for hybridization by digestion or shearing, gives random fragments that may not match the gene targets on the array as well as cDNA. The sheared/digested DNA varies in length and the longer fragments may contain pieces of several genes.

The common analysis of aCGH data focuses on the so-called log-ratio *M*_*i *_= log_2_(*S*_*i*_/*I*_*i*_) where *S*_*i *_is the signal intensity of the sample strain and *I*_*i *_similar for the index strain, for gene *i *[2]. A small log-ratio indicates a weak sample strain signal, and hence the gene is most likely divergent. Using a t-test statistics or a modified regularized t-test statistics as for example offered by the SAM [8] is no practicable alternative for this kind of experiments, as in practise there are mostly no replicate measures at all. Hence, statistical analysis is limited to finding a high quality diagnostic score which can be used for a ranking of candidate divergent genes. This is the reason for focussing on scores which can be calculated from two signal intensities alone, the photomultiplier intensity readouts for the labels from index and sample strain respectively. A fixed cutoff on the log-ratio axis, separating divergent from present genes, is most likely sub-optimal due to the variation inherent in microarray experiments. As a consequence, it seems mostly impossible to learn an optimal fixed cutoff as classifier from a training data set even in the rare cases where such data set would be available. Discriminant analysis approaches will therefore fail in the typical case. It seems more appropriate to determine such a cutoff dynamically from the data set in question for each analysis. To deal with this [9] introduced a method for calculating a dynamical cutoff from the log-ratio distribution. Considering the histogram in Figure 5, it is natural to assume that the heavy left tail of the distribution is due to divergent genes. Based on this assumption [9], suggested a calculation of the cutoff somewhere around the transition between the body and the left tail of the sample distribution. The data analysis tool developed from this approach is GACK [10].

We will in this paper extend and formalize the idea of [9], to combine it with the data rotation approach by [11]. This allows us to use both the information inherent in the distribution of the log-ratios as well as that about the V-shaped patterns in the MA-plot, as observed by [11]. Finally, from estimated probabilities for each gene to be absent or present in the sample strain, we calculate what we call the ROTMIX score for each gene. We use a set of simulated data sets as well as a set of experimental data sets from fully sequenced sample strains to validate the usefulness of the ROTMIX score for ranking and classification.

## Results

### Analyses of experimental data sets

In order to test and compare our approach to the conventional use of log-ratios as well as the rotation approach suggested by [11] we performed experiments where sequences of both sample and index genomes were known *a priori*. In a normal experiment only the sequence of the index strain is known, but this design provides us with data where aCGH analysis results can be validated by direct sequence comparisons.

**Figure 1:**
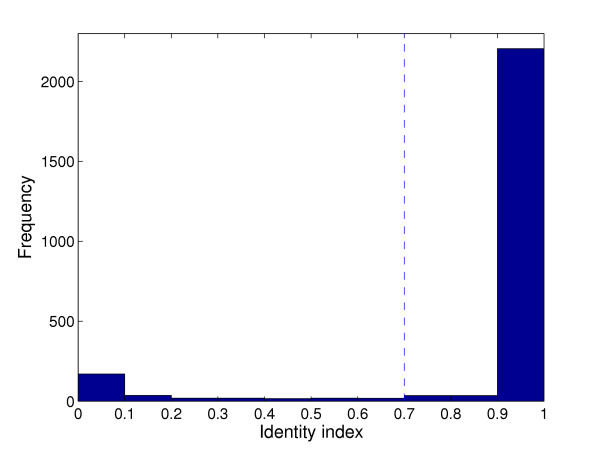
**Identity index histogram**. A histogram of the identity index for each gene in the data set COL versus N315. The identity index tend to be either very close to 0 or 1. The marked threshold at 0.7 is used to separate divergent from present genes unless otherwise stated. The histograms for the other three data sets are very similar, see [16].

A list of truly divergent genes is essential to validate the proposed method. For experimental data sets no such list exists with absolute certainty, even for fully sequenced genomes. However, from the sequence data it is evident that there exist two natural groups of genes, either as present or divergent in the sample strain. Figure 1 shows a histogram of the identity indices from the BLAST searches in one data set. Genes with identity index below 0.7, or other cutoff if stated, are treated as divergent.

A classifying score's ability to discriminate divergent from present genes in a given data set can be summarized in a receiver operating characteristic (ROC) curve [12]. The trade-off between sensitivity and specificity is captured by the area under curve (AUC) statistic, where a large AUC (close to 1) indicates a good separation of the classes. Table 1 summarizes AUC-values, where we have compared the ranking using the ROTMIX-score to the ranking by log-ratio *M *or rotated log-ratio M* from Equation 4.

**Table 1:** Results of ROC analysis. The area under the ROC-curve (AUC) in each data set. Genes have been ranked according to the ROTMIX-score, and by log-ratios *M *or rotated log-ratios M*.

Ranking variable	COL vs N315	COL vs Mu50	TIGR4 vs R6	TIGR4 vs G54
${\hat{\rho }}_{i}$	0.91	0.83	0.84	0.82
*M*_*i*_	0.73	0.62	0.84	0.79
$M_{i}^{*}$	0.90	0.80	0.79	0.78

**Figure 2:**
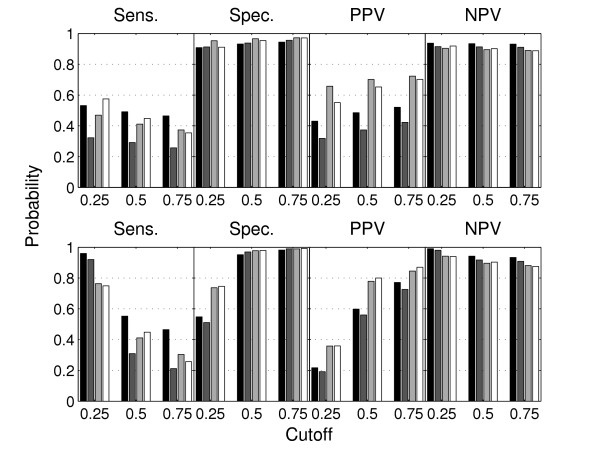
**Varying classification cutoff**. The effect of varying the classification cutoff. The bars mark different data sets, COL versus N315 (black) and Mu50 (dark gray), TIGR4 versus R6 (light gray) and G54 (white). In the upper panels classifications are done using the log-ratio based posterior probability, and in the lower panels the ROTMIX-score.

A ROC curve deals with sensitivity and specificity, which are estimates of *P*($\hat{C}$ = 0|*C *= 0) and *P*($\hat{C}$ = 1|*C *= 1), respectively, for a given data set. The area under the ROC-curve indicates a variable's potential for classification, but the problem remains to actually pick a cutoff. Figure 2 shows specific values for sensitivity and specificity for three different cutoffs. Once a classification has been done, it will in most cases also be natural to consider *P*(*C *= 0| $\hat{C}$ = 0) and *P*(*C *= 1|$\hat{C}$ = 1) in addition to sensitivity and specificity. Their corresponding estimates from a given data set we denote Positive Predictive Value (PPV) and Negative Predictive Value (NPV), and these are also included in Figure 2.

**Figure 3:**
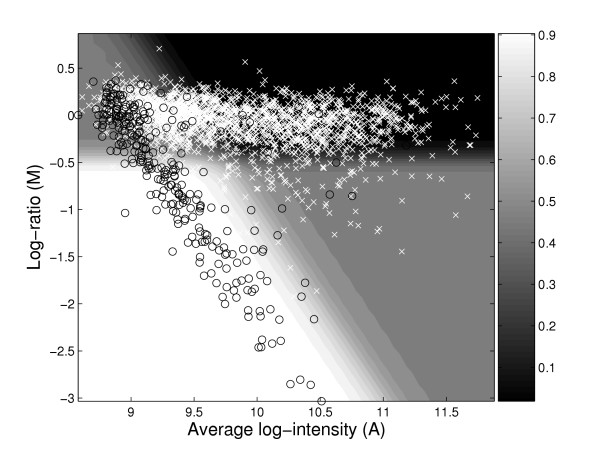
**Heatplot of ROTMIX in the MA-plane**. ROTMIX-classification in the data set COL vs. N315 in the *MA*-plane. Divergent and present genes are marked by black circles and white crosses, respectively. The underlying shading illustrates how the ROTMIX-score varies over the plane, numerical values given by the gray-scale bar at the right. The ROTMIX score is an average of two posterior probabilities using plain and rotated data, for details refer to equation 6 in the Methods part.

Figure 3 is an illustration of how the ROTMIX-score separates genes in an MA-plot. There are three major zones. The white zone is where genes will clearly be classified as divergent and the black zone clearly as present. The gray zone is a 'doubt' zone, and classification in this zone will depend largely on the choice of classification cutoff.

### Analyses of simulated data sets

A set of 1000 simulated experiments for different random seeds was the basis for comparing the conventional, data-rotation and ROTMIX approaches. In the case of the conventional approach, increasing normalized *M*-values were used as score to rank genes as candidates for divergent genes, whereas for the data rotation approach increasing M* values were used (Equation 4), and for the ROTMIX analysis, $\hat{\rho }$ values (Equation 6).

**Figure 4:**
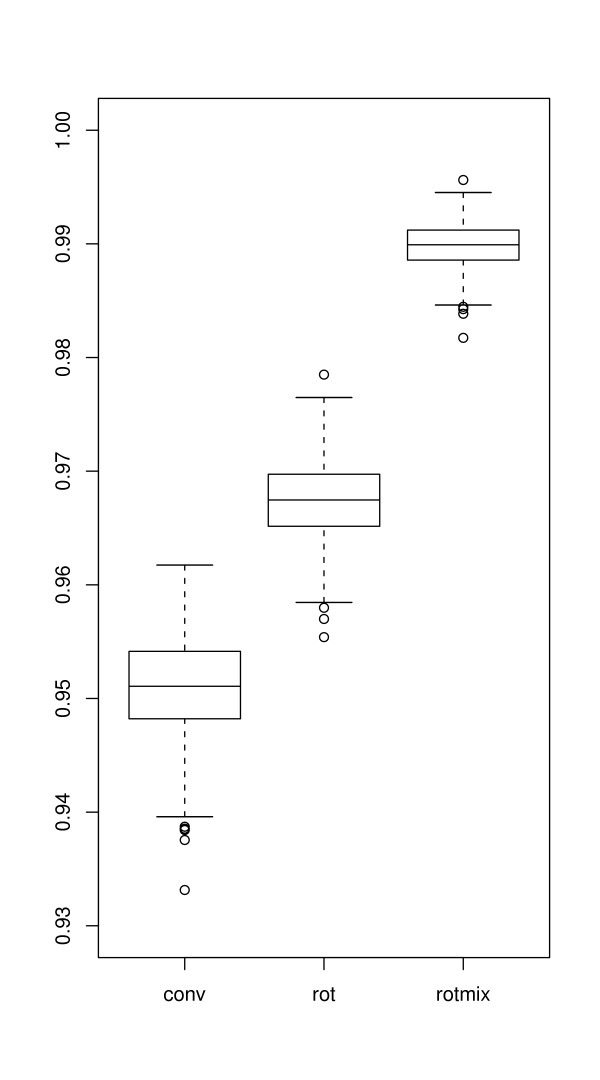
**Simulation results**. A box-plot showing the distributions of AUC values for 1000 evaluated simulated data sets with each of conventional analysis, data rotation, and ROTMIX analysis. The boxes indicate the median and upper and lower quartile. The whiskers indicate additional 1.5 interquartile range on each side, and the small circles indicate extreme results outside this range.

For each data set and each of the three analysis approaches; conventional, data rotation and ROTMIX approach, the ranking score for each gene was taken as a possible cutoff and rates of true positives, true negatives, false positives and false negatives recorded to construct ROC-curves and AUC-values (Figure 4).

**Figure 5:**
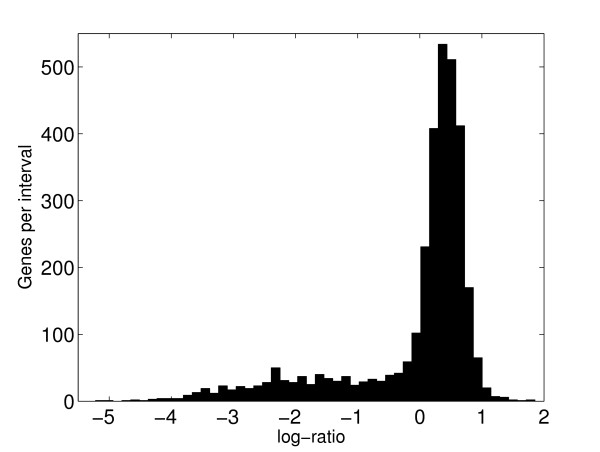
**Typical histogram of microbial aCGH log-ratios**. A typical histogram of log_2_-ratios for a microbial aCGH experiment. The data are for *Enterococcus faecalis*, index strain V583 against sample strain MB143.

## Discussion

Array based CGH is a high-throughput biotechnology that is consolidating itself as a useful tool in microbial comparative genomics. Despite many applications of this technology in analyzing genome-genome similarity, there is no real consensus on how to analyze the data and draw conclusions from the experiments. We have in this paper suggested an efficient method for ranking genes, and subsequently classifying them into two groups, present and divergent. Essentially we calculate a diagnostic score as the average posterior probability of divergence from the mixture model in (2) when fitted to the data before and after the rotation. We have demonstrated its usefulness for simulated validation data as well as for four different hybridizations with only fully sequenced sample strains. Results were compared to two other proposed analysis approaches.

Log-ratio based ranking is by far the most common in papers dealing with microbial aCGH data. In some microbial aCGH analyses the cutoff log-ratio separating divergent and present genes is held constant at -1.0 (or 1/2 for ratios) [13,14]. Others use a cutoff relative to the distribution of all data, e.g. [15], who treated all genes with log-ratio more than 2 standard deviations below the overall mean as putative deletions. Log-ratio based ranking is also the fundament for [9] and the data analysis tool GACK [10]. An alternative way of ranking was introduced by [11], using the data rotation. From Figure 1 as well as from Table 1 it seems that the ROTMIX-score separates divergent from present better than the two other ways of ranking genes. In all cases, the AUC-value for the ROTMIX-score is as good or better than the other two. The differences are, however, small, and based on only four independent experimental validation sets, the differences are not significant. The simulations, however, indicate a stable difference since every ROTMIX-result is better than all other results. The AUC-values for the ROTMIX-score are comparatively high, ranging from around 0.8 to well over 0.9 depending on data set and identity threshold.

Our experimental validation data are from experiments with fully sequenced strains, but still there is some degree of uncertainty regarding which genes are truly divergent. We have based our analysis on nucleotide sequence identity, since this is what a microarray can measure. Using an identity threshold of 0.7 gives 12–16% divergent genes, which is a likely number, compared with other aCGH studies [2]. We have performed analyses with other choices of identity threshold (0.5–0.9), and the results are similar to those in Table 1 (see [16]).

When we interpret the classifying variable as a posterior probability of divergence (or presence) a natural cutoff for classifying divergent and present genes is 1/2. Figure 3 illustrates the effects of different cutoffs. For the log-ratio based classifications (upper panels) there is little effect of a varying cutoff within the range shown. This is because when fitting a two-component gaussian mixture to data like those in Figure 5 the major peak will give a rather narrow density describing the present genes, i.e. almost all genes will have a posterior probability of divergence very close to 0 or 1. From the lower panels of Figure 3 we notice that the results of the ROTMIX-classification is sensitive to a varying cutoff. As expected, a gradually increased cutoff will produce higher PPV and specificity but lower sensitivity. This means that if a gene has a large ROTMIX-score it is also more likely to be divergent.

Fixing the cutoff at 1/2 gives a significant improvement of PPV for the ROTMIX case compared to the log-ratio classification in the upper panels (p-values below 0.05 for all data sets in a significance test of proportions). This translates to a relevant reduction in the false discovery proportion for the genes ranked first using the ROTMIX-score. Thus, time and costs for subsequent proving low-throughput experiments are considerably lowered. For sensitivity and NPV there is no significant difference, and for specificity ROTMIX gives a significant, but in practice not important, improvement.

Using a score-based approach rather than an established statistics like for example a t-test or a regularized modification thereof (e.g. SAM, [8]) is necessary because of the usual absence of replicate measures in aCGH screenings. Moreover, a mixture model score is more robust against normalization problems. Any t-test like statistic, using a null hypothesis of equal signals for index and sample genome, would classify all genes with significant deviation in signals as divergent. The mixture fit, however, searches for two distributions of signals, and there is no need to assume that present genes always produce equal signals in the sample and index strain.

MA-plots should always accompany any analysis using the ROTMIX-procedure. It is in this space the ROTMIX-procedure operates, as illustrated in Figure 4. It is of course essential that the MA-plot graph more or less has the V-shaped form. If not, the ROTMIX-score may give dubious results, but of all microbial aCGH data sets we have seen, a majority has this characteristic pattern. As more and more bacterial strains are sequenced, we will see more multi-genome arrays in the future. From such arrays we can also detect genes that are present in the sample strain but not in the index strain. Following the reasoning behind the data rotation of [11], we expect such genes to be found around a line of slope +2 in the MA-plot. Experiments on such arrays could also be analyzed by our procedure, with some natural adjustments, given that such data show a corresponding W-shaped pattern. We have seen some data confirming this, but more research should be done before we can be conclusive.

The current validation data indicate that the most severe problem faced is the rather low sensitivity, (between 0.4 and 0.6, see Figure 3) when using a classification cutoff around 1/2. This is not surprising, since divergent genes are in general grossly outnumbered by present. Future efforts should, however, probably focus on this. One approach could be to make better use of extra information sources. We are actually facing a classification problem, but with no training data available. A partial training of the classifier could however be done using genes known to be present, i.e. the core minimal genome genes [17]. Experiments with cDNA microarrays still lacks the repeatability needed to transfer actual parameter estimates from one experiment to another. To achieve this, highly specialized arrays are required [18], at high costs and reduced versatility.

## Conclusion

We have devised an efficient, sensitive and specific procedure for detecting divergent genes from microbial aCGH experiments. A simple procedure based on gaussian mixture models and data rotation provides a score for each gene, which is an average of two posterior probabilities of divergence. When tested on simulated data as well as on four different experimental validation data sets with only fully sequenced strains, this ROTMIX-score seems to be an improvement of the standard log-ratios for ranking and classifying genes into divergent and present.

## Methods

### Pre-processing and conventional analysis

Data acquisition and preprocessing is as in cDNA microarray experiments, except for the normalization step. Most normalization procedures have an underlying assumption of (locally) symmetric distribution of log-ratios. In expression experiments this is usually an acceptable assumption, but as seen from Figure 5, clearly not for microbial aCGH data. All experimental data sets we consider are from dye-swap experiments with multiple spots (three or four) for each gene on each array. We have therefore implemented a normalization procedure essentially similar to the 'self-hybridization' suggested by [19].

Ranking genes according to the normalized log-ratio corresponds to the conventional approach for analyzing aCGH data.

### Mixture model

Classifying genes of the index strain as present or divergent with respect to a sample strain is not a typical classification problem, as training data in the narrow sense are not available for every single experiment. On the other hand, some knowledge about the log-ratios of the divergent genes is available, and we try to make use of this prior knowledge in our proposed analysis. We build our analysis upon a two-component mixture model framework.

Let *C*_*i *_be the class variable for gene *i*, i.e.

$C_{i}=\{\begin{array}{ll} 0 & \text{if gene }i\text{ is divergent}\\ 1 & \text{if gene }i\text{ is present} \end{array}\;\left(1\right)$

The unconditional probability of gene *i *being divergent is *P*(*C*_*i *_= 0) = *π*. Let *M*_*i *_be the observed log-ratio, or some transformation of it (see below), for gene *i*. For divergent genes we assume this log-ratio is distributed according to the density *f*_0_(*M*), and similar, *f*_1_(*M*) is the density for present genes. Thus, the joint density *f*_*C, M *_(*C*, *M*) is defined, and its marginal in *M *is the mixture model

*f*_*M*_(*M*_*i*_) = *πf*_0_(*M*_*i*_) + (1 - *π*)*f*_1_(*M*_*i*_)     (2)

From the joint and marginal density we also get the conditional density *f*_*C*|*M *_(C_*i*_|*M*_*i *_= *f*_*C, M *_(*C*_*i*_, *M*_*i*_)/*f*_*M *_(*M*_*i*_). The posterior probability of divergence for gene

*i*, *P*(*C*_*i *_= 0|*M*_*i*_) = *p*_0_(*M*_*i*_) is then given by this density as

$p_{0}\left(M_{i}\right)=\frac{\pi f_{0}\left(M_{i}\right)}{\pi f_{0}\left(M_{i}\right)+\left(1-\pi \right)f_{1}\left(M_{i}\right)}\;\left(3\right)$

Assuming *f*_*k *_~ *N*(*μ*_*k*_, $\sigma_{k}^{2}$), *k *= 0,1, all parameters can be estimated from Equation (2) without any knowledge of *C*_*i*_. Either maximum likelihood estimation using the EM-algorithm or a Bayesian approach using the Gibbs-sampler will do this job satisfactory [20].

### Data rotation

In our novel analysis approach, we are aiming at combining the conventional analysis together with the data rotation approach [11].

**Figure 6:**
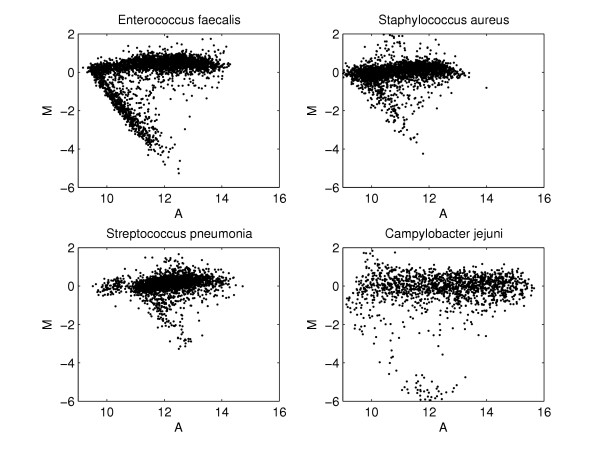
**MA-plots for different aCGH experiments**. Plots of log-ratio (*M*) against average log-intensity (*A*) for four aCGH data sets from four different bacteria. The characteristic V-shaped pattern is most clearly visible in the upper left panel, but is also more or less present in the other MA-plots.

The data rotation approach is based on the presumption that divergent genes will tend to populate around a line of slope -2 when their log-ratio (*M*) is plotted against their average log-intensity (*A*). The observation of a V-shaped pattern in the MA-plots for microbial data sets is common (Figure 6). The lower 'arm' of this V will in general have a slope of -2, which is explained as follows:

Each of the two intensities obtained per gene can be seen as a combination of two components

*S*_*i *_= *b*_*i *_+ ${S^{\prime }}_{i}$

*I*_*i *_= *b*_*i *_+ ${I^{\prime }}_{i}$

where *b*_*i *_is some baseline intensity due to non-specific hybridization, and ${S^{\prime }}_{i}$ and ${I^{\prime }}_{i}$ are signal intensities for sample and index strain, respectively. The baseline intensity is expected to be small if compared to a signal for a gene present in the index strain. For divergent genes, ${S^{\prime }}_{i}$ should ideally be zero, while ${I^{\prime }}_{i}$ must still be expected to be comparatively large. Thus, for divergent genes *S*_*i *_≈ *b*_*i *_and *I*_*i *_≈ ${I^{\prime }}_{i}$, and we get

*M*_*i *_= log(*S*_*i*_/*I*_*i*_) ≈ log(*b*_*i*_) - log(${I^{\prime }}_{i}$)

*A*_*i *_= (log(*S*_*i*_) + log(*I*_*i*_))/2 ≈ $\frac{1}{2}$ (log(*b*_*i*_) + log(${I^{\prime }}_{i}$))

and hence *M*_*i *_≈ 2 log(*b*_*i*_) - *2A*_*i*_. This suggests that divergent genes should, when plotting *M*_*i *_versus *A*_*i*_, propagate around some line with slope -2.

As proposed by [11], we will use a rotation of the axes (*A*, *M*) → (A*, M*) as described by the linear map

$\left[\begin{array}{c} M^{*}\\ A^{*} \end{array}\right]=X\left[\begin{array}{c} M\\ A \end{array}\right]\;\left(4\right)$

where

$X=\left[\begin{array}{cc} \sin\left(\gamma \right) & -\cos\left(\gamma \right)\\ \cos\left(\gamma \right) & \sin\left(\gamma \right) \end{array}\right]\;\left(5\right)$

and where *γ *= arctan(-2).

**Figure 7:**
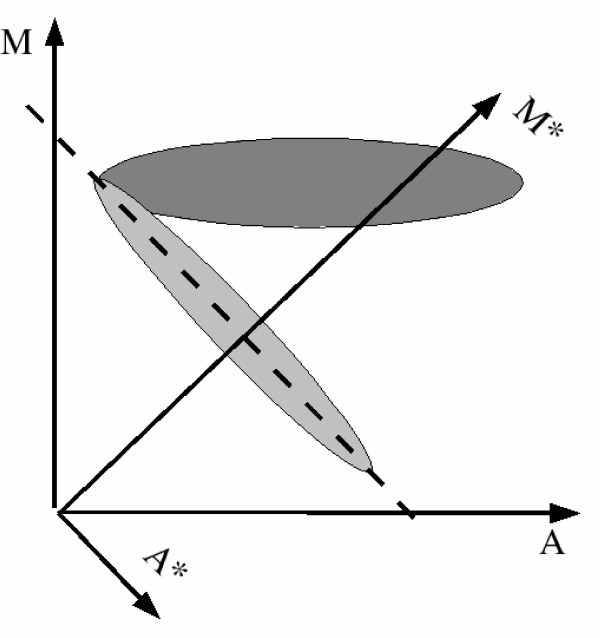
**The data rotation**. An illustration of the data rotation. Present (dark gray) and divergent (light gray) genes are presumed to group themselves into a V-shaped pattern in the MA-plot. The divergent genes populates along a line of slope -2 (broken line). After rotation the A* axis will be parallell to this line, and genes can be ranked according to their M*-value.

Ranking genes by their M*-value rather than by their *M*-value gives an alternative way of separating divergent from present, as illustrated in Figure 7. In [11] a reduction in false positives is reported as the main advantage of this procedure.

### The ROTMIX-score

To further improve the classification, we propose a ranking of the genes according to a score which is the average posterior probability of divergence from (3) when fitting the mixture model to both *M *and M* values, respectively.

First, fit the two-component gaussian mixture model from Equation (2) to the log-ratios, and let $C_{i}=\{\begin{array}{ll} 0 & \text{if gene }i\text{ is divergent}\\ 1 & \text{if gene }i\text{ is present} \end{array}\;\left(1\right)$ (*M*_*i*_) be the estimated posterior probability of divergence for gene *i *found from Equation (3). The density describing the major peak of the data, *f*_1_, is very well estimated in this case. The divergent genes are, however, most likely smeared out over a large range of log-ratios, and *f*_0 _is probably not very well approximated. This may lead to genes with very large log-ratios having a large ${\hat{p}}_{0}$ (*M*_*i*_) if ${\hat{f}}_{0}$ is very wide. To avoid this artifact we require that

${\hat{p}}_{0}\left(M_{i}\right)=\underset{j}{\min}{\hat{p}}_{0}\left(M_{j}\right),\forall j:M_{j}>{\hat{\mu }}_{1}$

where ${\hat{\mu }}_{1}$ is the estimated location of *f*_1_.

Second, we perform the data rotation from Equation (4) and fit the two-component gaussian mixture to the rotated log-ratios $M_{i}^{*}$. The mixture estimation can be based on all data, but as suggested by [11], we use a truncated data set, where only genes having log-ratio smaller than ${\hat{\mu }}_{1}$ from the first mixture model, are used. In this truncated data set the peak of the presumably divergent genes is more pronounced and hence easier to estimate. Nevertheless, this gives us another set of estimates ${\hat{p}}_{0}$($M_{i}^{*}$) for every gene. In this case *f*_1 _may be poorly estimated, and hence, we make a similar requirement as we did for the first estimates

${\hat{p}}_{0}\left(M_{i}^{*}\right)=\underset{j}{\max}{\hat{p}}_{0}\left(M_{j}^{*}\right),\forall j:M_{j}^{*}<{\hat{\mu }}_{0}$

and ${\hat{\mu }}_{0}$ is the estimated location of *f*_0_.

Finally, the ROTMIX-score is the average of the two estimates

${\hat{\rho }}_{i}=({\hat{p}}_{0}(M_{i})+{\hat{p}}_{0}(M_{i}^{*}))/2\;\left(6\right)$

### Classification

We classify genes based on the ROTMIX-score, using a cutoff between 0 and 1. A natural choice is 1/2, which is according to the Bayes rule [21], but other choices may be made. This means $\hat{C}$ = 0 if the probability is larger than the cutoff and $\hat{C}$ = 1 otherwise. The choice of cutoff will depend on the focus of the analysis. If we are primarily searching for divergent genes, e.g. looking for characteristic divergent regions on the chromosome, it is probably wise to choose a larger cutoff to avoid too many false positives (genes misclassified as divergent). On the other hand, if the focus is on the present genes, e.g. estimating the minimum genome over all strains, we would naturally avoid false negatives (genes misclassified as present), and choose a smaller cutoff. We could also introduce a doubt-zone, i.e. only classify genes who are below a lower threshold or above an upper.

### Data

#### Experimental data

In order to compare aCGH analysis approaches we conducted aCGH experiments with fully sequenced strains, i.e. both index and sample strains' gene contents are available as gold standards. Microarrays for *Staphylococcus aureus *index strain COL, were used in aCGH analyses against strains Mu50 and N315 and similar for *Streptococcus pneumoniae *index strain TIGR4 against R6 and G54. Full genomes as well as identified gene sequences for these strains can be downloaded from the Comprehensive Microbial Resource (CMR) at TIGR [22], the *Streptococcus *strain G54, is available at the Spanish National Cancer Centre [23].

Due to allele differences, silent mutations and possible sequence errors in the databases we cannot expect a gene from one strain to be found with exact similarity in another strain even if it is truly the same gene. High hybridization signals are based on similarity at the nucleotide sequence level. To establish a quantification of this similarity, each index gene was locally aligned against a database consisting of the sample strain sequences for each experiment.

To reduce the element of randomness in the choice of BLAST parameters, we made several BLAST searches for every gene, keeping the match score constant at 1 and varying the remaining parameters systematically around their default values. In all cases the DUST low-complexity filter was turned off. For each search the best hit for index gene *i *was recorded, and an identity index was calculated as the number of exact matching residue-pairs divided by the number of residues in the index gene. The median identity index for gene *i*, was used as the identity-score for that gene.

For a chosen threshold we predicted gene *i *to be divergent if the corresponding identity index is below this threshold and present otherwise. Unless otherwise stated, in the downstream analysis we used the threshold 0.7 to establish a list of divergent genes from each data set.

#### Simulated data

In addition to using the experimental data sets for purpose of methods comparison, we also used a set of simulated data sets according to [24] and [11]. The underlying model is

*S*_*i *_= *α*_*S *_+ *β*_*S*_*X*_*Si *_· exp(*u*_*i *_+ *v*_*Si*_) + *e*_*i *_+ *w*_*Si*_

*I*_*i *_= *α*_*I *_+ *β*_*I*_X_*Ii *_· exp(*u*_*i *_+ *v*_*Ii*_) + *e*_*i *_+ *w*_*Ii*_

together with the following variable explanations and parameter settings: *S*_*i *_and *I*_*i *_denotes simulated measured fluorescence intensity for gene *i *from the sample and index strain, respectively. Each simulated experiment consisted of 3000 genes, where *i *= 1,..., *n*_div _were divergent. We modeled scenarios for different proportions of divergent genes, 0.05 ≤ *n*_div_/3000 ≤ 0.5. *X*_*Si *_and *X*_*Ii *_model the true values for the expected fluorescence signals. Intensities of present and absent genes are

$\begin{array}{l} \log_{2}\left(X_{Si}\right)\sim \{\begin{array}{cc} N\left(5,0.25\right) & \text{when }i\leq n_{\text{div}}\\ N\left(8,0.25\right) & \text{when }i>n_{\text{div}} \end{array}\\ \log_{2}\left(X_{Ii}\right)\sim N\left(8,0.25\right) \end{array}$

i.e. expected sample intensities for divergent genes are modeled with 12.5% of the sample intensity of present genes.

Moreover, fixed background parameters are

*α*_*S *_= *α*_*I *_= 300

*β*_*S *_= *β*_*I *_= 0.5.

Remaining quantities are gaussian variables with zero expectation and variance equal to 0.25, chosen as recommended by [24] and resulting in simulated data with similar distributions of data points in the MA-plot as in our laboratory experiences. The random variables are interpreted as in [11]: *u*_*i*_, *v*_*Si *_and *v*_*Ii *_are multiplicative error terms, *u*_*i *_models the gene-specific effects and *v*_*Si *_and *v*_*Ii *_the multiplicative gene-dye-interactions, *e*_*i*_, *w*_*Si *_and *w*_*Ii *_refer to additive errors.

## Authors' contributions

LS and DR have contributed equally to this work, by discussing and polishing ideas, programming in Matlab and R, and writing the manuscript.

LN has done the validation experiments, under supervision of ÅA, who has also introduced the problem in the first place, and been the supplier of arrays and cultures for the experiments.

AZ and AA have been discussion partners and supervisors for the statistical part.

## References

[B1] Fitzgerald JR Sturdevant DE Mackie SM Gill SR Musser JM Evolutionary genomics of Staphylococcus aureus: Insight into the origin of methicillin-resistant strains and the toxic shock syndrome epidemic Proceedings of the National Academy of Science 2001 98 8821 8826 10.1073/pnas.161098098 PMC37519 11447287

[B2] Dorrell N Champion OL Wren BW Application of DNA Microarrays for Comparative and Evolutionary Genomics Methods in Microbiology 2002 33 121 136

[B3] Pinkel D Segraves R Sudar S Clark S Poole I Kowbel D Collins C Kuo W Chen C Zhai Y Dairkee S Ljung B Gray JW Albertson DG High resolution analysis of DNA copy number variation using comparative genomic hybridization to microarrays Nature Genetics 1998 20 207 211 10.1038/2524 9771718

[B4] Pollack JR Perou CM Alizadeh AA Eisen MB Pergamenschikov A Williams CF Jeffrey SS Botstein D Brown PO Genome-wide analysis of DNA copy-number changes using cDNA microarrays Nature Genetics 1999 23 41 46 10.1038/14385 10471496

[B5] Fridlyand J Snijders AM Pinkel D Albertson DG Jain AN Hidden Markov models approach to the analysis of array CGH data Journal of Multivariate Analysis 2004 90 132 153 10.1016/j.jmva.2004.02.008

[B6] Jong K Marchiori E Meijer G van der Vaart A Ylstra B Breakpoint Identification and Smoothing of array Comparative Genomic Hybridization data Bioinformatics Advanced Access 2004 16 1 2

[B7] Autio R Hautaniemi S Kauraniemi P Yli-Harja O Astola J Wolf M Kallioniemi A CGH-Plotter: MATLAB toolbox for CGH-data analysis Bioinformatics 2003 19 1714 1715 10.1093/bioinformatics/btg230 15593402

[B8] Tusher VG Tibshirani R Chu G Significance analysis of microarrays applied to the ionizing radiation response PNAS 2001 98 9 5116 5121 10.1073/pnas.091062498 11309499 PMC33173

[B9] Kim CV Joyce EA Chan K S F Improved analytical methods for microarray-based genome-composition analysis Genome Biology 2002 3 11 research0065.1 0065.17 10.1186/gb-2002-3-11-research0065 12429064 PMC133449

[B10] The GACK software http://falkow.Stanford.edu/whatwedo/software/software.html.

[B11] Repsilber D Mira A Lindroos H Andersson S Ziegler A Data rotation improves genomotyping efficiency Biometrical Journal 2005 47 4 585 598 10.1002/bimj.200410160 16161813

[B12] Hanley JA McNeil BJ The meaning and use of the area under a receiver operating characteristic (ROC) curve Radiology 1982 143 29 36 7063747 10.1148/radiology.143.1.7063747

[B13] Björkholm B Lundin A Sillén A Guillemin K Salama N Rubio C Gordon JI Falk P Engstrand L Comparison of Genetic Divergence and Fitness between Two Subclones of *Helicobacter pylori* Infection and Immunity 2001 2001 7832 7838 10.1128/IAI.69.12.7832-7838.2001 PMC98879 11705965

[B14] Dorrell N Mangan JA Laing KG Hinds J Linton D Al-Ghusein H Barrell BG Parkhill J Stoker NG Karlyshev AV Butcher PD Wren BW Whole Genome Comparison of *Campylobacter jejuni *Human Isolates Using a Low-Cost Microarray Reveals Extensive Genetic Diversity Genome Research 2001 11 1706 1715 10.1101/gr.185801 11591647 PMC311159

[B15] Behr MA Wilson MA Gill WP Salamon H Schoolnik GK Rane S Small M Comparative Genomics of BCG Vaccines by Whole-Genome DNA Microarray Science 1999 284 1520 1523 10.1126/science.284.5419.1520 10348738

[B16] Supplementary material http://arken.umb.no/~larssn/bioinformatics/ROTMIX/

[B17] Gil R Silva FJ Peretó J Moya A Determination of the Core of a Minimal Bacterial Gene Set Microbiology and Molecular Biology Reviews 2004 518 537 10.1128/MMBR.68.3.518-537.2004 15353568 PMC515251

[B18] Dunman PM Mounts W McAleese F Immermann F Macapagal D Marsilio E McDougal L Tenover FC Bradford PA Petersen PJ Projan SJ Murphy E Uses of *Staphylococcus aureus *GeneChip in Genotyping and Genetic Composition Analysis Journal of Clinical Microbiology 2004 4275 4283 10.1128/JCM.42.9.4275-4283.2004 15365023 PMC516287

[B19] Yang YH Dudoit S Luu P Speed T Normalization for cDNA Microarray Data http://www.stat.berkeley.edu/users/terry/zarray/Html/normspie.html

[B20] McLachlan GJ Peel D Finite Mixture Models 2000 New York: John Wiley & Sons

[B21] Ripley BD Pattern Recognition and Neural Networks 1996 Cambridge: Cambridge University Press

[B22] The Institute of Genomic Research http://www.tigr.org/.

[B23] Spanish National Cancer Centre http://bioinfo.cnio.es/data/Spneumo/.

[B24] Cui X Kerr MK Churchill GA Data transformations for cDNA microarray data Statistical applications in genetics and molecular biology 2003 2 article 4 10.2202/1544-6115.1009 16646782

